# Molecular Simulation of the Separation of Isoleucine Enantiomers by β-Cyclodextrin

**DOI:** 10.3390/molecules24061021

**Published:** 2019-03-14

**Authors:** Elena Alvira

**Affiliations:** Departamento de Física, Universidad de La Laguna, 38202 La Laguna, Tenerife, Spain; malvira@ull.edu.es; Tel.: +34-922-318-258

**Keywords:** cyclodextrins, isoleucine, enantiomers, interaction energy, molecular mechanics, molecular dynamics, inclusion complex, elution order

## Abstract

Molecular mechanics and dynamics simulations were carried out to study the capacity of isoleucine enantiomers to form inclusion complexes with β–cyclodextrin, and to be discriminated by this chiral compound, in vacuo and with different solvents. Solvents were characterized not only by the value of dielectric constant ε in the Coulombic interaction energy, but also by the neutral and zwitterion configurations of isoleucine. Whereas the discrimination between the enantiomers for ε ≤ 2 is due to the electrostatic contribution, these differences are mainly due to the Lennard-Jones potential for ε > 2. The most enantioselective regions are located near the cavity walls, independently of the solvent. D-Ile is more stable than L-Ile in broader regions in vacuo, but L-Ile presents more stable locations with water. Isoleucine can form inclusion complexes with β–cyclodextrin in vacuo and with different solvents. Two probable configurations are deduced from the molecular dynamics simulation, in which the guest is always inside the cavity and with the carboxylic end of the amino acid oriented towards either rim of β–CD. In the simulation, the enantiomers preferentially occupy regions with greater chiral discrimination. The first eluted enantiomer in vacuo and with different solvents is L-Ile, independently of the solvent polarity.

## 1. Introduction

Cyclodextrins (CDs) are composed of glucose units (six for α–CD, seven for β–CD, eight for γ–CD, etc) forming macrocyclic molecules resembling truncated cones, whose cavities with different internal diameters are able to include molecules of different dimensions and configurations [1,2,3]. The capacity of CDs and derivatized CDs for catalysis and chiral recognition of racemic compounds, is due to their ability to form host-guest inclusion complexes. For this reason, CDs have extensive applications in the manufacture of pharmaceutical, textile, pesticides, food or aroma products [4]. Isoleucine (Ile) is an α–amino acid present in most common proteins and it is an isomer of leucine. It is one of nine essential amino acids in humans and necessary to liver, muscle tissues and fats, but it cannot be synthesized by the body, so it must be ingested. Inclusion complex formation and chiral separation of amino acids and their derivatives by CDs have been studied with different experimental techniques such as electrospray mass spectrometry, capillary electrophoresis and gas chromatography [5,6]. In particular, the inclusion complex formation of L-Ile zwitterion with both α–and β–CD, with 1:1 stoichiometry is confirmed by nuclear magnetic resonance (NMR) techniques, surface tension and conductivity measurements [7]. The enantiodiscrimination and inclusion complex formation of several amino acids with β–CD in gas phase has also been analysed by molecular dynamics simulations [8], where it was determined that Ile enantiomers can locate almost totally inside the cavity in the complexes with minimum energy, and L-Ile is the first eluted enantiomer. 

Previously, we studied the enantiodiscrimination and formation of inclusion complexes of several amino acids with β–CD by means of molecular mechanics (MM) and dynamics (MD) simulations. Since we studied alanine (Ala), valine (Val) and leucine (Leu) in vacuo and with solvents like water, we considered two configurations for the amino acids, non-polar and zwitterion [9,10,11,12]. Furthermore, some variations were introduced in the model applied to each amino acid to improve the simulation method. We showed that they were able to form β–CD inclusion complexes with water and other solvents but not in vacuo, diverging from the results proposed by Ramirez et al. [8]. The aim of the present study is to theoretically examine the interaction between Ile enantiomers and β–CD in vacuo and with different solvents. New modifications were introduced in the molecular simulation, among them the ab initio method used to determine the amino acid configurations, instead of the force field proposed by Weiner et al. for the molecular mechanics simulation of nucleic acids and proteins [13,14], used in our former studies.The most important result, and thereby the main difference obtained with respect to the other amino acids, is the capacity of Ile to form inclusion complexes with β–CD in vacuo. The conclusions of the present study agree with those proposed by Ramirez et al., not only that related to the inclusion complex formation, but also the lowest energy structure of the complexes and the elution order in the separation of Ile enantiomers. The main results and discussion of molecular mechanics and dynamics simulations are presented in Section 2, where the interaction energies between β-CD and Ile are evaluated to investigate the formation of inclusion complexes and the chiral discrimination of enantiomers. The potential energy and simulation model utilized are described in Section 3.

## 2. Results and Discussion

### 2.1. Molecular Mechanics Simulation

**Figure 1:**
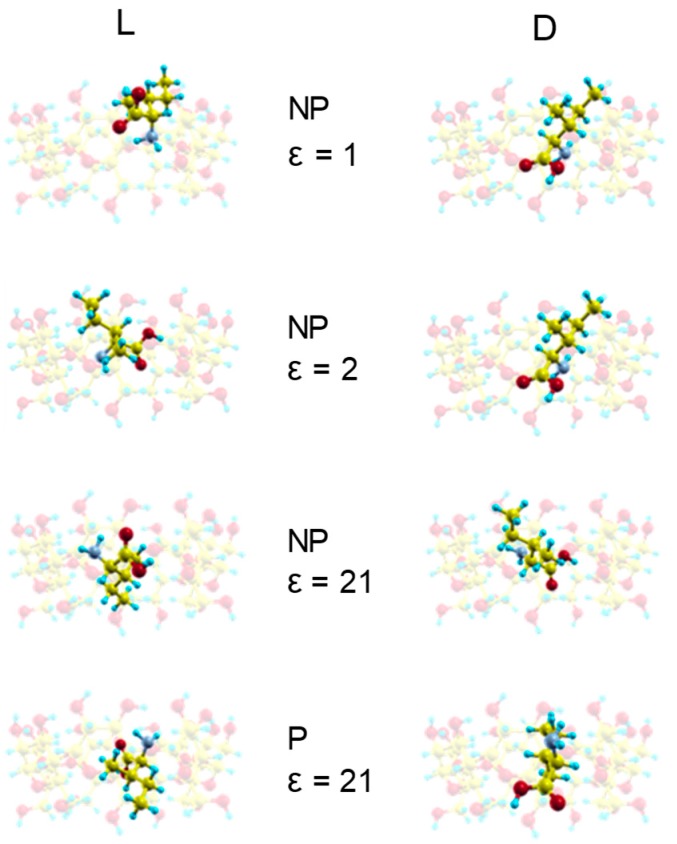
The complex configurations with minimum interaction energies *E_min_* for ε = 1 (NP structure), ε = 2 (NP structure), ε = 21 (NP structure) and ε = 21 (P structure). NP and P structures are neutral and zwitterion configurations respectively. The guest molecule has been superimposed for clarity.

The interaction energy *E_total_* between Ile enantiomers and β–CD is determined by MM in vacuo and also with different solvents like hydrocarbons, benzene, toluene, acetone, ethanol and water. Two configurations for the amino acid (neutral or zwitterion) are considered to simulate the solvent polarity, along with different values of dielectric constant ε in the electrostatic contribution to the interaction energy *E_total_*. The *E_min_* is the lowest value of *E_total_* and the complex configuration of a system calculated by MM is the host-guest configuration of energy *E_min_*, which can indicate an inclusion complex if the guest molecule is located totally or partially inside the cavity. Previously, we studied the interaction between β–CD and some amino acids (Ala, Val and Leu) with the mentioned solvents, and we found that the lowest energy structures were inclusion complexes except in vacuo and for ε = 2 [9,10,11,12]. However, the minimum energy complexes formed between Ile enantiomers and β–CD are inclusion complexes (Figure 1) [15], both in vacuo and with solvents. The guest molecule has been superimposed in Figure 1 for clarity, but it is always located inside the cavity. The discrepancy between the results obtained for Ile and the other amino acids may be due to the ab initio method used in the present study to determine the guest configuration, and the factors influencing this difference will be the subject of the next study. In addition, the lowest energy structures formed in vacuo agree with those proposed by Ramirez et al. [8], not only for the inclusion complex formed, but also for the configuration itself, because the carboxylic end of the amino acid is oriented towards the small rim of the cavity. The orientation of D-Ile in the inclusion complexes formed with solvents is the same as in vacuo, and the guest centre of mass is located near the cavity centre. The orientation of L-Ile in the complex formed in vacuo is different, but there is another structure with an energy 0.9% greater than the minimum, whose configuration (as in ε = 2) also agrees with that proposed by Ramirez et al. The guest centre of mass is located near the wider rim of β–CD for L-Ile in vacuo and for ε = 2, but it is located near the cavity centre in solvents with ε > 2. In these, the carboxylic end is oriented towards the wider rim of β–CD. The solvent polarity does not influence the inclusion complex configurations.

The minimum interaction energy (*E_min_*) and its different contributions are included in Table 1. The Lennard-Jones term is always the main contribution to *E_min_* and its value is similar in those cases in which the centre of mass of the amino acid is also similar (Figure 1), the differences due to the guest orientation being insignificant. The complex in vacuo shows the greatest electrostatic energy *E_ele_* and this contribution decreases when ε increases. In non-polar solvents with high dielectric constants, *E_ele_* is positive although its magnitude is trivial. The H-bond term only contributes to *E_min_* in the complexes where the guest orientation allows the formation of this type of bonds, and its magnitude in these cases is about −0.5 kcal/mol. The angle bending *E_angle_* makes the greatest contribution to the intramolecular energy and its greatest variations with different solvents is seen in L-Ile (about 38%), the difference in *E_torsion_* being about 0.05% for each enantiomer. However, the elution order cannot be determined from the minimum energy because the guest does not always adopt the corresponding configuration, as seen later in the molecular dynamics simulation. 

The intermolecular energy *E_inter_* calculated by MM is represented by the potential energy surface and the penetration potential *W* (the curve joining the minimum intermolecular energy for every plane Z = constant). The energy is always deeper inside than outside the cavity, which constitutes an attractive force to include the guest into β–CD. 

**Figure 2:**
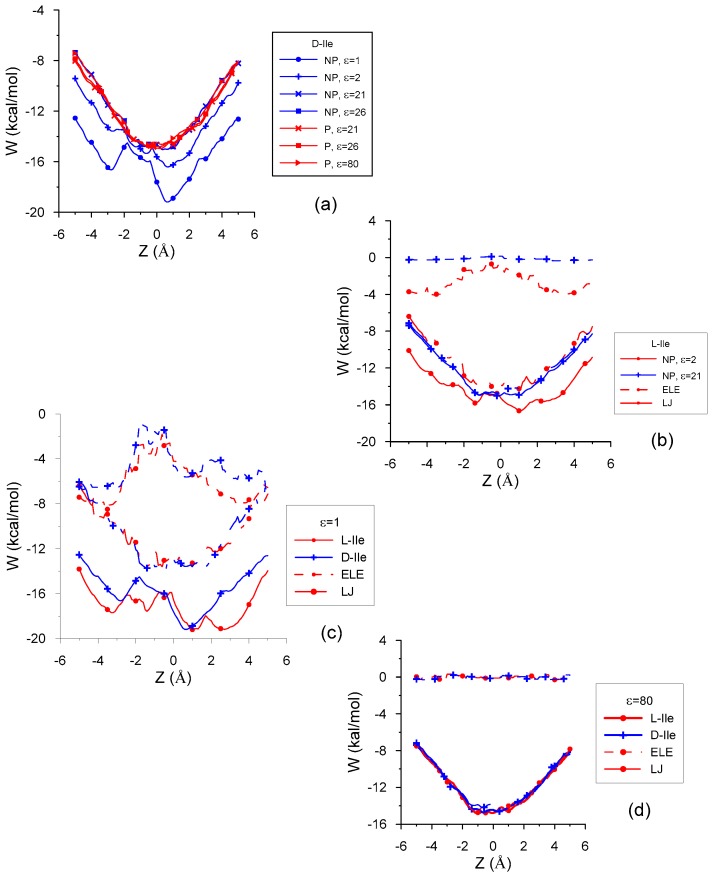
The penetration potential *W* and its different contributions for the interaction between β–CD and (**a**) D-Ile in vacuo and with different solvents; (**b**) L-Ile in non-polar solvents; (**c**) Ile enantiomers in vacuo; (**d**) Ile enantiomers with water.

Figure 2a represents the penetration potential *W* for the interaction between D-Ile and β–CD in vacuo and with different solvents. There are two cases clearly different from the rest (ε = 1 and ε = 2); these differences are due not only to the deeper minima of *W*, but mainly because *W* does not seem to be a well potential. Figure 2a also shows the centre of mass position of minimum energy in every case. The main contribution to *W* is the van der Waals contribution (represented by a Lennard-Jones potential, LJ), as shown in the interaction between L-Ile and β–CD in non-polar solvents (Figure 2b), and is nearly the same inside the cavity for these solvents. The LJ potential does resemble a well potential inside the host, whose minimum value is located near the cavity centre. The electrostatic energy (ELE) is nearly constant inside the cavity for greater values of ε and does not depend on the solvent polarity. It decreases when ε increases, amounting to even positive values in some solvents although its influence is negligible compared to the other contributions to *W* (Figure 2b,d). The minimum value of ELE for ε = 1 and ε = 2 corresponds to positions of the guest centre of mass outside the cavity, near the small rim of β–CD, and it increases inside the cavity. This contribution appears to be a potential barrier whose height for ε = 1 is double that for ε = 2. As a consequence, *W* presents two relative minima for these ε, also separated by a potential barrier, higher for ε = 1. Figure 2c shows *W*, LJ and ELE energies for L- and D-Ile in vacuo. The greater differences in *W* between enantiomers correspond to positions of the guest centre of mass near the wide rim of β–CD, and the ELE term is the greatest contribution to these differences (3 kcal/mol in vacuo and 2 kcal/mol for ε = 2). Therefore, the discrimination between the Ile enantiomers is due to the ELE energy for ε ≤ 2, not only because this term contributes to *W* up to 40% inside the cavity, but it also presents the greatest difference between the values of L- and D-Ile. These differences for ε > 2, independently of solvent polarity, can reach 0.8% (Figure 2d) and are mainly due to the LJ potential, because this term contributes most to the intermolecular energy (about 95%) with these ε.

**Figure 3:**
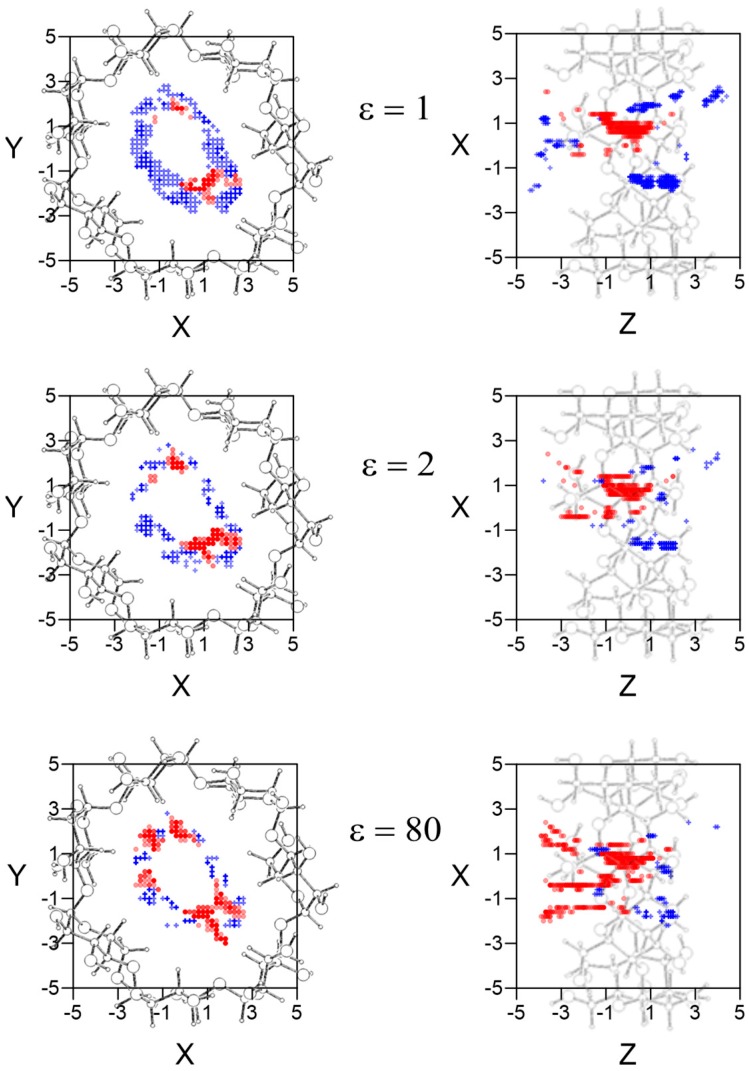
The projections in XY and XZ planes of the points of potential surfaces with greater differences in energy, red circles correspond to locations in which L-Ile is more stable and blue crosses those for D-Ile. The bolder the symbol, the greater difference in energy it represents. A schematic representation of the projections of β–CD is included in those planes.

The *W* potential represents the minimum intermolecular energy through the cavity, but the guest does not always adopt the orientation of minimum energy in each location when it moves inside and outside β–CD. Therefore the potential energy surface for each enantiomer is determined at each grid point by the average Boltzmann energy for different guest orientations, instead of the lowest energy [16,17]. The most enantioselective regions for the different values of ε are inside the β–CD, and there are regions where L-Ile is more stable than D-Ile and vice versa. In order to clearly represent the results, Figure 3 shows the projections in XY and XZ planes of the points of potential surfaces with greater differences in energy, red circles correspond to locations in which L-Ile is more stable and blue crosses those for D-Ile. The bolder the symbol, the greater difference in energy it represents. A schematic representation of the projections of β–CD is included in those planes. The most enantioselective regions are located near the cavity walls, independently of the solvent, at grid points where the LJ potential approaches repulsive values. D-Ile is more stable than L-Ile in broader regions in vacuo; however the stability of enantiomers changes when ε increases, independently of the solvent polarity and L-Ile occupies more extensive stable locations than D-Ile with water (ε = 80).

### 2.2. Molecular Dynamics Simulation

**Figure 4:**
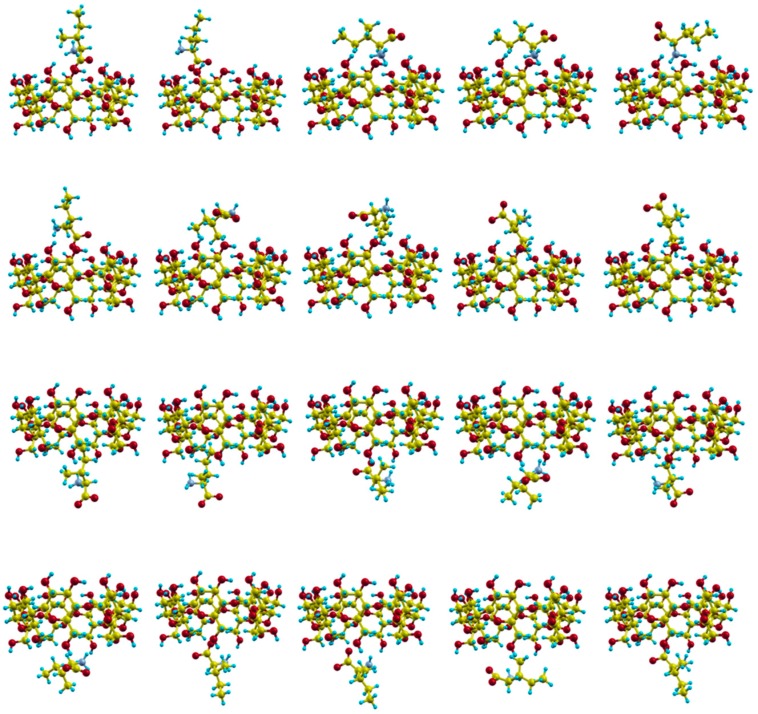
The initial centre of mass positions and orientations for the amino acid in the trajectories.

The movements of the molecules due to their mutual interactions are simulated in MD by trajectories, i.e., the consecutive centre of mass positions and orientations of the molecules during the simulation time. Different initial values for molecular velocities and dispositions (centre of mass position and orientation) are considered to represent some of the conditions occurring in the process of inclusion complex formation. We calculated 20 trajectories with random initial velocities and orientations for the amino acid, and in which the guest centre of mass is located outside the cavity, in front of both rims of β–CD (Figure 4).

The enantiomers usually enter the cavity, move around inside during a certain period of time (Table 2) forming a stable complex (residence time *t*) and then exit from the CD, although they do not always pass through the cavity. However, the Ile enantiomer does not form an inclusion complex in some trajectories (external trajectories) in which it continues moving around the host, tending to move away. The initial centre of mass position of the guest influences the MD simulation because there are more trajectories starting from the narrow rim of β–CD, which are transformed into external trajectories, independently of the initial guest orientation. When the enantiomer is initially oriented parallel to the rims of CD or with the methyl chain pointing towards the cavity, it tends to remain outside. The evolution of Ile also depends on solvent polarity because the zwitterions do not form inclusion complexes in trajectories starting from the narrow rim, with the carboxylic end of the amino acid pointing towards the cavity. Therefore, the factors that mainly influence the trajectories, and therefore, the possibility of inclusion complex formation, are the solvent and the initial conditions of Ile, so we consider the same values for both enantiomers. The ability of CDs to separate enantiomers is based on their forming inclusion complexes, therefore in the simulation of each enantiomer we consider only those trajectories in which they remain totally or partially inside the cavity, during the residence time *t* (Table 2). The number of trajectories contributing to the simulation of each enantiomer is always smaller than 20 and depends on the solvent, although the influence of solvent polarity on this number is negligible. Table 2 also shows the mean value of the binding free energy *F* for each enantiomer in vacuo and with different solvents. This energy depends on the movements of the guest in each trajectory and is thus mainly influenced by the possibility of inclusion complex formation. *F* decreases when ε increases, the complex formed by Ile with β–CD in vacuo being the most stable. The solvent polarity influences the energy *F*, because with the same value of ε the complexes formed by zwitterions are more stable than the neutral configurations of amino acid. The first eluted enantiomer in vacuo is L-Ile (Table 2), in agreement with the experimental and theoretical evidence provided by Ramirez et al. [8]. We obtained the same elution order for the different solvents. The greatest capacity of β–CD to discriminate the Ile enantiomers occurs in vacuo, because the difference in energy is also the greatest. Table 2 shows the average residence time *t_mean_* for each enantiomer in the simulation, which indicates the mean time each enantiomer remains inside β–CD. The values of *t* vary from hundreds of ps to the simulation time (5 ns), although this residence time does not reflect the capacity of inclusion complex formation because there are trajectories in which the guest spends very different *t* inside the cavity. There are also trajectories in which the enantiomer does not enter the cavity with the same *t* as another in which it does, although in general the time Ile remains close to β–CD is shorter when it moves outside the cavity. The elution order and the enantiomer with least mean residence time are in agreement except for ε = 26 (NP). 

**Figure 5:**
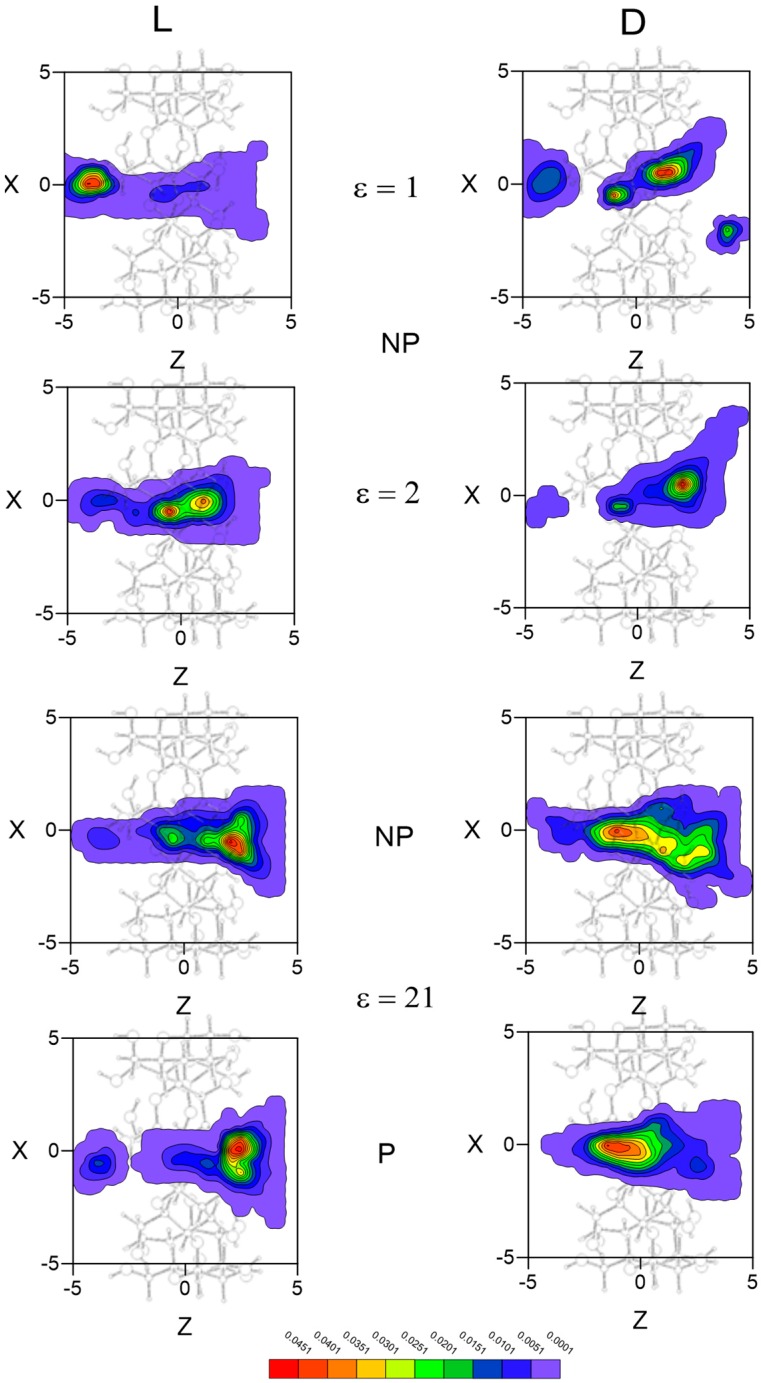
The projections in the XZ plane of the regions with greatest probability of presence for Ile in vacuo and with different solvents. A schematic projection of β–CD is included in this plane.

The inclusion complex configurations for the enantiomers are determined in MD according to the most probable guest orientations corresponding to the preferred centre of mass positions. The regions with greatest probability of presence for Ile are located inside the cavity, although the preferred configuration depends on the enantiomer and solvent. Whereas the preferred location of L-Ile in vacuo is near the small rim of β–CD, the centre of mass position of this enantiomer tends towards the centre or the wide rim of the cavity with any solvent (Figure 5). The greatest probability of presence for D-Ile in vacuo is at the wide rim of β–CD, but this probability moves towards positions between the centre and the small rim of β–CD when ε increases. Solvent polarity does not influence the probability of presence in an inner position, whereas the results for Ile are smaller and distributed over larger areas when ε increases. The enantiomers preferably remain in favourably enantioselective regions, due to the greater differences in the interaction energy with β–CD.

**Figure 6:**
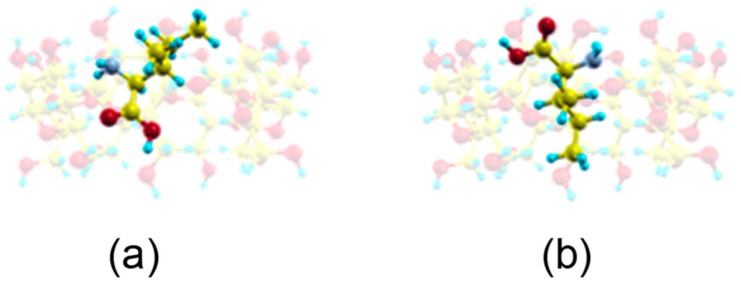
The most probable inclusion complex configurations for Ile enantiomers in vacuo deduced from the MD simulation, in which the carboxylic end of the amino acid is pointing towards (**a**) the narrow rim of β–CD; (**b**) the wide rim of β–CD. The guest molecule has been superimposed for clarity.

The guest orientation in these highly probable zones does not remain constant, but rather varies continually. However, the sizes of host and guest do not allow Ile to move freely inside the cavity so as to adopt the minimum energy configuration. These sizes also impede the enantiomer rotating an angle of 180° with respect to the cavity axis inside the β–CD. This rotation only occurs outside the β–CD before entering the cavity. From the MD simulation we have obtained two probable configurations for the inclusion complex (Figure 6a,b) [15], in which the carboxylic end of the amino acid is pointing towards either rim of β–CD. The guest molecule has been superimposed in Figure 6 for clarity, but it is located inside the cavity. These two configurations are deduced from the most probable guest orientations supported by the preferred centre of mass positions, which are the same for both enantiomers in vacuo and with solvent. There is no relation between the initial guest disposition and the most probable inclusion complex configuration deduced from a trajectory. The structure represented in Figure 6a is similar to the lowest energy structure proposed by Ramirez et al. for Ile enantiomers in vacuo.

## 3. Materials and Methods 

### 3.1. Molecular Mechanics Simulation

The interaction potential *E_total_* between Ile enantiomers and β–CD is modelled by the sum of the intramolecular *E_intra_* and intermolecular *E_inter_* energies, as proposed by Weiner et al. for molecular mechanics simulation of nucleic acids and proteins [13,14]. The intramolecular energy indicates the conformational changes in the host and guest, and is modelled by a sum of the torsional energy, stretching and bending vibrations of bonds. The intermolecular energy represents the interaction between non-bonded atoms and is determined by a sum of the van der Waals (Lennard-Jones potential), Coulombic and H-bond contributions:

(1) $$\begin{array}{c} E_{total}=\sum_{i<j}\left[\frac{A_{ij}}{R_{ij}^{12}}-\frac{B_{ij}}{R_{ij}^{6}}+\frac{q_{i}q_{j}}{\varepsilon R_{ij}}\right]+\sum_{H-bonds}\left[\frac{C_{ij}}{R_{ij}^{12}}-\frac{D_{ij}}{R_{ij}^{10}}\right]+\sum_{bonds}k_{r}{\left(r-r_{eq}\right)}^{2}\\ +\sum_{angles}k_{\theta }{\left(\theta -\theta_{eq}\right)}^{2}+\sum_{dihedrals}\frac{V_{n}}{2}\left[1+\cos\left(n\phi -\gamma \right)\right] \end{array}$$

where *r* represents bond lengths, θ bond angles, *ϕ* torsional angles of molecules and *R_ij_* the distance between the *i*th atom of Ile and the *j*th atom of the host. The molecular configuration of β–CD, its net atomic charges [18] and the AMBER force field parameters (*A_ij_*, *B_ij_*, *C_ij_*, *D_ij_*, *k_r_*, *r_eq_*, *k_θ_*, θ*_eq_*, *V_n_*, *n*, γ) are taken from the literature [13,14]. The new torsion potentials of the modified AMBER ff99SB protein force field (AMBER ff99SB-ILDN) are considered for the two dihedral angles of Ile (N-C^α^-C^β^-C^γ^) [19]. The atomic coordinates of Ile are calculated by the Hartree-Fock method, using the 6-31G** basis set implemented in the MOLPRO package [20,21], instead of the force field modelled by Weiner et al. [13,14] that we previously used in the simulations of some amino acids [9,10,11,12]. Two molecular configurations and atomic charge distributions of the amino acid are proposed to represent the non-polar structure (NP) and the zwitterion (P), in which the amino and carboxyl radicals of amino acids are replaced by NH_3_^+^ and COO^−^ [9,10,11,12]. Therefore all atoms are considered in the simulation because the two configurations of Ile differ in the positions of several hydrogen atoms. These positions can contribute decisively to the formation of H-bonding between host and guest, and this may be reflected in the interaction energy *E_total_*. Different solvents are represented by the dielectric constant ε in the Coulombic term of *E_total_* and the amino acid structure, supposing ε = 1 and a non-polar configuration for Ile, to simulate the interaction in vacuo. In this study we also examine several values of ε with both structures of the amino acid; in this way the influence of solvent polarity on the simulation can be analysed (Table 1).

The reference system is located over the principal axis of the β–CD and the origin of the space-fixed frame at the centre of mass of the CD. The *Z* axis is located on the cavity axis, thus the *XY* plane is parallel to the rims of β–CD. The relative position of Ile enantiomers with respect to the absolute reference system is defined by the guest centre of mass position and orientation, given by the Euler angles. The method applied to study the interaction between Ile and β–CD is the same previously used with several molecules: the energy *E_total_* is calculated for different orientations and positions of the guest centre of mass inside and outside the CD [9,10,11,12]. The complex configuration of Ile with β–CD in vacuo and with different solvents is determined from MM as the position and orientation of the guest in the absolute minimum energy *E_min_*. This minimum value is obtained calculating *E_total_* by Equation (1) for different orientations (about 23,000), at each grid point (−5 ≤ *X* ≤ 5, −5 ≤ *Y* ≤ 5, −5 ≤ *Z* ≤ 5) at which the distance between two consecutive points is 0.1 Å. The minimum value of *E_total_* for the molecular orientations is selected at each grid point. The results given by the MM simulation provide the potential energy surfaces (PES), complex configurations, penetration potential (*W*), the minimum value of *E_total_* (*E_min_*) and its different contributions. The complex configuration obtained from the simulation is the host-guest configuration of *E_min_*, and it is considered an inclusion complex if the molecule of Ile has its structure completely or partially included inside the cavity. The penetration potential *W* is defined as the curve connecting the minimum value of intermolecular energies for every plane Z = constant, and shows the change in *E_inter_* when its path through the cavity is non axial. Since Ile does not always adopt the minimum energy orientation while moving near the cavity, due to the separation process, the PES is calculated from the average Boltzmann energy at each grid point, instead of the lowest energy obtained for the different orientations of Ile [16,17].

### 3.2. Molecular Dynamics Simulation

The particle trajectories are determined in MD simulation by solving the classical equations of motion, due to the mutual interaction between Ile and CD. These equations represent both the translational motion of the guest centre of mass, which depends on the total force acting on the body, and the rotation about its centre of mass due to the total applied torque. The problem of divergence in the orientational equations is solved by using four quaternions related to the Euler angles, and the resultant equations involve their first derivatives, the angular velocities and their time derivatives for each molecule. To integrate numerically the equations of motion, different initial conditions of the molecule of Ile are considered to determine the trajectories: velocities, centre of mass position and orientation (guest disposition). The values of the initial velocities (translational and rotational) are calculated as a consequence of the temperature of the process (293 K), and their direction is determined randomly. All these values are the same for both enantiomers, to avoid the influence of factors that artificially introduce differences in the simulation, other than their interaction with the host. However, the initial atomic positions of enantiomers can never be the same because they are mirror images. Several criteria were previously selected to minimize the effect of the initial disposition of amino acids on chiral discrimination: the Geometric criterion, in which the average atomic distance between the initial configurations of enantiomers is minimized; the Numeric criterion where the initial centre of mass position and orientation for enantiomers are the same values; and the Energetic criterion in which the difference in the intermolecular energy between each enantiomer and the host is minimized [11,12]. In the present study we determined the initial disposition of Ile in the trajectories, minimizing simultaneously the average atomic distance and the interaction energy between each enantiomer and β–CD. These magnitudes obtained in different trajectories varied from 0.19 to 4.15 × 10^−2^ (Å) for the average atomic distance, and from 4.77 × 10^−7^ to 1.43 × 10^−5^ (kcal/mol) for the energy. The simulation time *t_s_* for each trajectory was 5 ns with a step of 1 fs, and the configuration and energies (kinetic and potential) were registered every 100 steps. When the initial centre of mass position of Ile was located outside the β–CD near the cavity walls, the guest does not enter the cavity, it stayed moving around the host and tended to move away. When the starting position of the enantiomer in the simulation was located near the cavity rims, it tended to enter the CD, remain inside for some period (residence time *t*) as a stable complex, and then move away from the CD. Therefore the equations of motion were finally integrated when the Ile molecule was located outside the cavity, in positions where its interaction with the β–CD was not attractive enough to be included again in the cavity. We previously studied the separation of Ala, Val and Leu enantiomers by β–CD by means of an MD simulation [9,10,11,12], introducing some variations in the method to improve the results in each case. The method applied to Ile enantiomers also differs from the former in the number of trajectories, selection of initial enantiomer dispositions and simulation time. Twenty trajectories were calculated in the present study, ten starting from each rim of β–CD, in this way the contributions of different initial centre of mass positions of the guest were equally considered in the simulation. To assess the influence of the relative orientation between the molecules in the simulation, we also considered different initial orientations of Ile: parallel to the rim of β–CD or with one of its side chains (the *NH*_2_, *COOH*, methyl or ethyl groups) pointing towards the cavity (Figure 4) [22]. An in-house computer program written in Fortran was developed to integrate numerically the equations of motion, and to perform constant temperature molecular dynamics, a variant of the leap-frog scheme (proposed by Brown and Clarke) [23] was used to separately constrain the kinetic energies of molecular rotation and translation [24].

The results determined in each trajectory of the MD simulation were: the guest configuration (centre of mass and orientation) at each step, the binding free energy *F*, residence time *t*, and position probability density. The average values of *F* and *t* in the simulation were also obtained (*F_mean_*, *t_mean_*). Whereas the residence time represents the time during which Ile remains close to the host, a guest molecule is capable of forming inclusion complexes with β–CD when it has greater probability of locating its structure completely or partially inside the host. The capacity to form inclusion complexes is calculated by the position probability density, which represents the most probable position of the enantiomer in the simulation. This density is determined by dividing the number density in a volume element by the total value of possible centre of mass positions for the Ile molecule. The number densities of presence or number of guest positions in each volume element is obtained from a grid [16,17]. The elution order is calculated in MD as the difference between the values of *F_mean_* for each enantiomer ∆*F* = *F_L_* − *F_D_* [25,26,27]. *F* indicates if the complex formation is energetically favourable with respect to the reactants. It is determined in the simulation using the following expression:

(2) $$F=-k_{B}T\ln\left(\sum_{i}\exp\left(-W_{i}/k_{B}T\right)\right)$$

where *W_i_* is the energy of the complex during the trajectories, *T* the temperature of the process (293 K) and *k_B_* Boltzmann’s constant [26]. If ∆*F* > 0, D-Ile is more tightly bound and L-Ile is the first eluted enantiomer. In contrast, if ∆*F* < 0, L-Ile spends more time inside β–CD.

## 4. Conclusions

The inclusion complex formation and chiral separation of isoleucine enantiomers by β–cyclodextrin, in vacuo and with different solvents, is analysed in this study by molecular mechanics and dynamics simulations. The host-guest configurations with minimum interaction energy determined by MM are always inclusion complex configurations in vacuo and with solvents like water. In these complexes, the centre of mass of D-Ile is located near the cavity centre with the carboxylic end of the amino acid oriented towards the small rim of the cavity, in agreement with the lowest energy structures in vacuo proposed by Ramirez et al. The orientation of L-Ile in these complexes is similar for ε = 1 and 2, but with the carboxylic end of the amino acid oriented towards the wide rim of β–CD in solvents with ε > 2. The van der Waals term is the main contribution to the intermolecular energy and the angle bending contributes most to the intramolecular energy. The most enantioselective regions are located near the cavity walls and the stability of enantiomers changes when ε increases. D-Ile is more stable than L-Ile in broader regions in vacuo, but L-Ile presents more extensive stable locations with water than D-Ile. While the discrimination between the Ile enantiomers in vacuo and for ε = 2 is on the basis of their electrostatic contribution to the interaction energy, these differences for ε > 2 are mainly due to the Lennard-Jones potential.

The factors that mainly influence inclusion complex formation in MD are the solvent and the initial conditions of Ile. The binding free energy *F* for each enantiomer decreases when ε increases, the complex formed by Ile with β–CD in vacuo being the most stable. The solvent polarity influences the energy *F*, since with the same value of ε the complexes formed by zwitterions are more stable than the neutral configurations of the amino acid. The first eluted enantiomer in vacuo is L-Ile, in agreement with the results provided by Ramirez et al., the selectivity being the same for the different solvents. The greatest capacity of β–CD to discriminate the Ile enantiomers occurs in vacuo, because the difference in energy is also the greatest. It can be concluded from the MD simulation that Ile can form two types of inclusion complexes with β–CD, in which the guest is always inside the cavity, and with the carboxylic end of the amino acid oriented towards either rim of β–CD.

There are discrepancies between our results obtained for Ile and those obtained previously in simulating the interaction between β–CD and other amino acids, the most important being those related to the inclusion complex formation in vacuo. Some of these differences are probably due to the method applied in the present study, which differs from the former in several variations such as the number of trajectories, simulation time and selection of initial dispositions of the enantiomers in the MD simulation. Since the MM simulation is not concerned with these values, it can be concluded that the results are mainly influenced by the ab initio method used in the present study to determine the guest configurations. Further studies applying the present model to Ala, Val and Leu can help to analyse the influence of these factors on the simulation.

## Figures and Tables

**Table 1:** The minimum interaction energy *E_min_* obtained with the AMBER force field for each enantiomer and the different contributions: Lennard-Jones *E_LJ_*, electrostatic *E_ele_*, hydrogen bonding *E_H-bond_*, bond stretching *E_bond_*, angle bending *E_angle_* and torsional energy *E_torsion_*.

Con.	ε	*E_min_* (kcal/mol)		*E_LJ_* (kcal/mol)		*E_ele_* (kcal/mol)		*E_H-bond_* (kcal/mol)		*E_bond_* (kcal/mol)		*E_angle_* (kcal/mol)		*E_torsion_* (kcal/mol)	
		L	D	L	D	L	D	L	D	L	D	L	D	L	D
NP	1	−10.91	−10.81	−11.89	−13.60	−7.38	−5.67	0.00	0.00	1.45	1.52	4.76	4.81	2.15	2.12
NP	2	−8.32	−8.02	−14.20	−13.87	−2.06	−2.61	−0.49	0.00	1.54	1.52	4.74	4.81	2.15	2.12
NP	21	−6.71	−6.70	−14.81	−14.68	0.13	0.02	−0.46	−0.49	1.57	1.57	4.71	4.76	2.15	2.12
NP	26	−6.73	−6.71	−14.81	−14.68	0.10	0.02	−0.46	−0.49	1.57	1.57	4.71	4.76	2.15	2.12
P	21	−8.32	−7.40	−14.80	−13.85	−0.43	−0.82	0.00	−0.49	1.49	1.43	3.27	4.21	2.15	2.12
P	26	−8.24	−7.29	−14.80	−14.30	−0.34	−0.28	0.00	−0.50	1.48	1.48	3.27	4.19	2.15	2.12
P	80	−8.09	−7.10	−14.93	−14.30	−0.08	−0.09	0.00	−0.50	1.52	1.48	3.25	4.19	2.15	2.12

**Table 2:** The number of trajectories, average binding free energy *F_mean_*, elution order and the average residence time *t_mean_* obtained for each enantiomer in the molecular dynamics simulation. *t*_s_ is the simulation time (5 ns).

Con.	ε	Number of Trajectories		*F_mean_* (kcal/mol)		Elution Order	*t_mean_* (ps)	
		L	D	L	D		L	D
NP	1	15	17	−9.77	−12.22	L	751.11	*t*_s_
NP	2	16	16	−9.45	−10.87	L	1098.18	*t*_s_
NP	21	16	17	−7.82	−8.17	L	659.62	675.35
NP	26	16	16	−7.48	−8.00	L	789.68	672.56
P	21	13	14	−8.84	−9.24	L	837.56	1212.11
P	26	14	18	−8.73	−9.18	L	554.58	1300.17
P	80	17	16	−8.67	−8.70	L	557.25	818.16
